# Integrated analysis of single-cell RNA-seq and chipset data unravels PANoptosis-related genes in sepsis

**DOI:** 10.3389/fimmu.2023.1247131

**Published:** 2024-01-03

**Authors:** Wei Dai, Ping Zheng, Jian Wu, Siqi Chen, Mingtao Deng, Xiangqian Tong, Fen Liu, Xiuling Shang, Kejian Qian

**Affiliations:** ^1^ Department of Intensive Care Unit, The First Affiliated Hospital of Jiangxi Medical College, Shangrao, Jiangxi, China; ^2^ Department of Clinical Medicine, Jiangxi Medical College, Shangrao, Jiangxi, China; ^3^ Department of Key Laboratory, Shanghai Pudong New Area People’s Hospital, Shanghai, China; ^4^ Department of Medical Technology, Jiangxi Medical College, Shangrao, Jiangxi, China; ^5^ Department of Intensive Care Unit, The First Affiliated Hospital of Nanchang University, Nanchang, Jiangxi, China; ^6^ The Third Department of Critical Care Medicine, Shengli Clinical Medical College of Fujian Medical University, Fujian Provincial Center for Critical Care Medicine, Fujian Provincial Key Laboratory of Critical Care Medicine, Fuzhou, China

**Keywords:** sepsis, Boruta algorithm, PANoptosis, single-cell RNA-seq, ssGSEA

## Abstract

**Background:**

The poor prognosis of sepsis warrants the investigation of biomarkers for predicting the outcome. Several studies have indicated that PANoptosis exerts a critical role in tumor initiation and development. Nevertheless, the role of PANoptosis in sepsis has not been fully elucidated.

**Methods:**

We obtained Sepsis samples and scRNA-seq data from the GEO database. PANoptosis-related genes were subjected to consensus clustering and functional enrichment analysis, followed by identification of differentially expressed genes and calculation of the PANoptosis score. A PANoptosis-based prognostic model was developed. *In vitro* experiments were performed to verify distinct PANoptosis-related genes. An external scRNA-seq dataset was used to verify cellular localization.

**Results:**

Unsupervised clustering analysis using 16 PANoptosis-related genes identified three subtypes of sepsis. Kaplan-Meier analysis showed significant differences in patient survival among the subtypes, with different immune infiltration levels. Differential analysis of the subtypes identified 48 DEGs. Boruta algorithm PCA analysis identified 16 DEGs as PANoptosis-related signature genes. We developed PANscore based on these signature genes, which can distinguish different PANoptosis and clinical characteristics and may serve as a potential biomarker. Single-cell sequencing analysis identified six cell types, with high PANscore clustering relatively in B cells, and low PANscore in CD16+ and CD14+ monocytes and Megakaryocyte progenitors. ZBP1, XAF1, IFI44L, SOCS1, and PARP14 were relatively higher in cells with high PANscore.

**Conclusion:**

We developed a machine learning based Boruta algorithm for profiling PANoptosis related subgroups with in predicting survival and clinical features in the sepsis.

## 1 Introduction

Sepsis is a pathology with a very high mortality rate in the critically-ill patients, which is caused by excess systemic uncontrolled inflammation and leading to the excessive discharge of inflammatory mediators (1). This results in various organ dysfunctions including cardiovascular, liver, pulmonary renal and brain impairments (2). As the latest sepsis guidelines suggest the impact of multiple organ dysfunction on the host, therefore, different types of cell death, including apoptosis (3), pyroptosis (4), and necroptosis (5) might be involved in sepsis.

Recently, the interaction between types of cell death has been found to have a significant impact on the development of sepsis. A recent review paper demonstrated that pyroptosis interacts with autophagy, apoptosis, NETosis, and necroptosis (4). Although all three types of cell death have been studies in sepsis, their communication pathways at the molecular level are largely unknown. Moreover, cell death mediated by apoptosis often occurs in heart, kidney, and other organ failures during sepsis (6) pyroptosis is more commonly associated with lethal sepsis (4, 7, 8). Necroptosis has also been observed in kidney damage resulting from mitochondrial dysfunction in sepsis (9). It is important to note that PANoptosis, a type of cell death widely reported in tumors (10) has not been studied in sepsis from a molecular level.

Our study aimed to fill this gap by examining PANoptosis-based molecular clustering and prognostic signatures to predict the immune landscape and prognosis in sepsis patients. We analyzed expression levels of PANoptosis-related genes (PANrgs) in 479 sepsis patients and identified three discrete PANoptosis clusters. Based on the PANscore derived from these clusters, patients were classified into two clusters, and a risk score was calculated. Using these data, we established a prognostic signature to predict overall survival (OS) in sepsis patients. We further mapped the hub genes in the risk model to single-cell RNA sequencing data.

## 2 Materials and methods

### 2.1 Data acquisition and preprocessing

Samples were downloaded from the GEO (https://www.ncbi.nlm.nih.gov/geo/) database using the GEOquery package of the R software (version 4.2.1, http://r-project.org/) from reliable sources. The samples in the sepsis expression profiling dataset GSE65682 dataset are all derived from Homo sapiens. GSE65682 is based on the GPL13667[HG-U219] Affymetrix Human Genome U219 Array platform. The data set contains 802 blood samples, including 760 cases of sepsis, and 479 cases of sepsis data were obtained after removing no clinical prognosis data. Datasets are standardized with annotated probes and other data cleaning operations. The clinical information of dataset GSE65682 will be used for clinical analysis. In GeneCard database (https://www.genecards.org/), PANoptosis-related genes were obtained by searching with PANoptosis as the keyword, and a total of 16 PANoptosis genes were obtained in combination with literatures (11–13) (**Supplementary Table 1**).

### 2.2 Consensus clustering based on PANoptosis-related genes

Consensus Clustering is a method that can determine the number and membership of possible clusters in a dataset (microarray gene expression). We used the “ConsensusClusterPlus” R package (14) to perform consensus clustering on the GSE65682 dataset using PANoptosis genes to better differentiate sepsis subtypes. We set the range for the number of clusters from 2 to 9 and repeated the process 100 times for stability, extracting 80% of the total sample each time. The clustering algorithm used was ‘k-means’ (clusterAlg = “km”), and the Euclidean distance was employed as the distance metric (distance=“euclidean”).

### 2.3 Functional enrichment analysis and gene set variation analysis

Gene ontology (GO) and pathway Kyoto Encyclopedia of Genes and Genomes (KEGG) enrichment analysis were performed on DEGs using clusterProfiler package (15), respectively. Single-sample gene set enrichment analysis (ssGSEA) was performed using the “GSVA” package to analyze the immune cell infiltration characteristics of each sample. Enrichment scores were calculated to represent relative expression in each sample.

### 2.4 Differentially expressed genes (DEG) analysis and calculation of PANoptosis score (PANscore)

To identify genes associated with PANoptosis, DEGs in different PANoptosis subtypes were determined using the limma R package. Important criteria were selected using adj.p < 0.05 and Fold change > 0.5. To evaluate the categorical value of DEG, dimensionality reduction was performed using the Boruta algorithm (16) and PANoptosis-related gene signatures were determined. Perform unsupervised clustering methods using the GSE65682 dataset. we applied hierarchical clustering with the ‘Ward.D2’ method and a ‘maximum’ distance. Patients were divided into different groups based on clustering of PANoptosis-related gene signatures for further analysis. PCA was then performed to determine the PANscore using principal components 1 and 2. PCA was conducted using the ‘prcomp’ function with scaling and centering of variables. This approach focuses on the score of the set containing the most significantly related genes and involves scaling down the scores of genes not tracked to other members of the set. The PANscore described according to a GGI-like procedure is calculated as follows: PANscore = ∑(PC1i + PC2i).

### 2.5 PANoptosis-related prognostic model based on PANscore

To assess the prognostic value of the PANscore in patients with sepsis, we first used the Kaplan-Meier method to look at the difference in survival between high and low PANscores. Subsequently, based on the expression profile data of GSE65682, using the survival package and the survminer package, the clinical factors and PANscore of GSE65682 were subjected to univariate and multivariate Cox regression analysis, and a multivariate Cox regression model was constructed to visualize the forest plot. Based on the multivariate Cox results, we used nomograms to assess the survival of patients with sepsis, and calibration and DCA curves to assess the reliability of the model. By combining various clinical information of sepsis patients, such as gender, age, pneumonia (community-acquired pneumonia (CAP) or hospital-acquired pneumonia (HAP)), thrombocytopenia, ICU-acquired infection (IAI), diabetes, etc. We analyzed the relationship between PANscore and them.

### 2.6 Animal maintaining and cell culture

Healthy male Sprague Dawley rats aged 8 weeks were obtained from the Animal Laboratory at Nanchang University and housed in sterilized cages under controlled conditions with a 45-55% relative humidity and a 12-hour light/dark cycle. Animals were acclimatized for one week prior to experimentation. All animal protocols were in accordance with the “Guidelines for the Care and Use of Experimental Animals” and received approval from the Institutional Animal Care and Use Committee of Nanchang University (Approval Number: 81960346). For the sepsis model, rats were anesthetized with an intraperitoneal injection of 50 mg/kg sodium pentobarbital and administered subcutaneous buprenorphine (0.05 mg/kg) every 6 hours post-operation for analgesia. A 1.5-2 cm midline abdominal incision was made to expose the cecum, which was then ligated at its distal end with a 4-0 suture, except for the sham group. A 21-gauge needle was used to puncture the cecum 1 cm distal to the ligation point, and the incision was subsequently sutured. All animals received 1 ml of pre-warmed (37°C) sterile saline for fluid resuscitation post-surgery. Venous blood samples were collected for further analyses.

Rat lung macrophage NR8383 cells and rat lung pulmonary epithelial type II cells RLE-6TN were seeded at a density of 106 in a 6-well plate containing 2 ml of medium per well, and when the cells grew to 50%, 1 μg/LPS stimulated cells with ml of LPS, LPS-treated cells and untreated NR8383 and RLE-6TN cells were collected after 24 hours respectively to establish a sepsis cell model and obtain cell specimens. Cell viability was determined by a CCK-8 assay measuring OD at 450 nm, providing a quantitative measure of cell health.

### 2.7 RNA extraction and real-time quantitative PCR

Total RNA was extracted using TRIzol reagent (Takara, Dalian, China). Reverse transcription was performed using Prime-Script RTase (Takara). Gene expression levels were determined by qPCR with the help of premix Ex-Taq (Takara) and normalized to GAPDH expression levels. We calculated expression levels using the 2^-ΔCT^ method.

### 2.8 Immunofluorescence experiments

Lung sections from mice were fixed in 4% paraformaldehyde, embedded in paraffin, and cut into 5-µm slices. After deparaffinization and antigen retrieval in citrate buffer, sections were blocked with 5% BSA. Primary antibodies were incubated overnight at 4°C, followed by fluorochrome-conjugated secondary antibodies. Nuclei were stained with 4’,6-diamidino-2-phenylindole (DAPI). Slides were visualized under a confocal microscope. Images were captured and analyzed using standardized imaging settings to ensure consistency. Experiments were performed in triplicate using standardized protocols.

### 2.9 Enzyme-Linked Immunosorbent Assay (ELISA)

The levels of specific proteins in the obtained samples were quantified using ELISA kits in accordance with the manufacturer’s instructions. Both samples and standards were processed in duplicate. The optical density (OD) was captured using a microplate reader set at a wavelength of 450 nm, with a reference wavelength of 630 nm to correct for any plate imperfections. Protein concentrations in the samples were extrapolated based on the standard curve using dedicated software for accurate quantification.

### 2.10 scRNA-seq dataset download and processing

We downloaded the sepsis single-cell sequencing sample GSE167363 from the GEO database and processed the data using the Seurat package, retaining cells with less than 10% mitochondrial genes, cells with a total number of genes greater than 200, and expression ranging from 200 to 12,000 and Genes expressed in at least 3 cells (17). The number of highly variable genes was set to 3000. The 10 samples were integrated by SCT correction. Then, dimensionality reduction of the data was performed using the tSNE method by setting the “DIMS” parameter to 16, and cell clustering was performed using the “KNN” method with the resolution set to 2. Subsequently, cells were labeled with various cell surface markers. Finally, import PANscore-related genes through the “PercentageFeatureSet” function to obtain the percentage of PANscore-related genes in each cell.

### 2.11 Statistical analysis

All data calculations and statistical analyses were performed using R programming. For the comparison of two groups of continuous variables, the statistical significance of normally distributed variables was estimated by the independent Student t test, and the differences among non-normally distributed variables were analyzed by the Mann-Whitney U test (ie, the Wilcoxon rank sum test). All statistical P values ​​were two-sided, with P < 0.05 considered statistically significant.

## 3 Results

**Figure 1:**
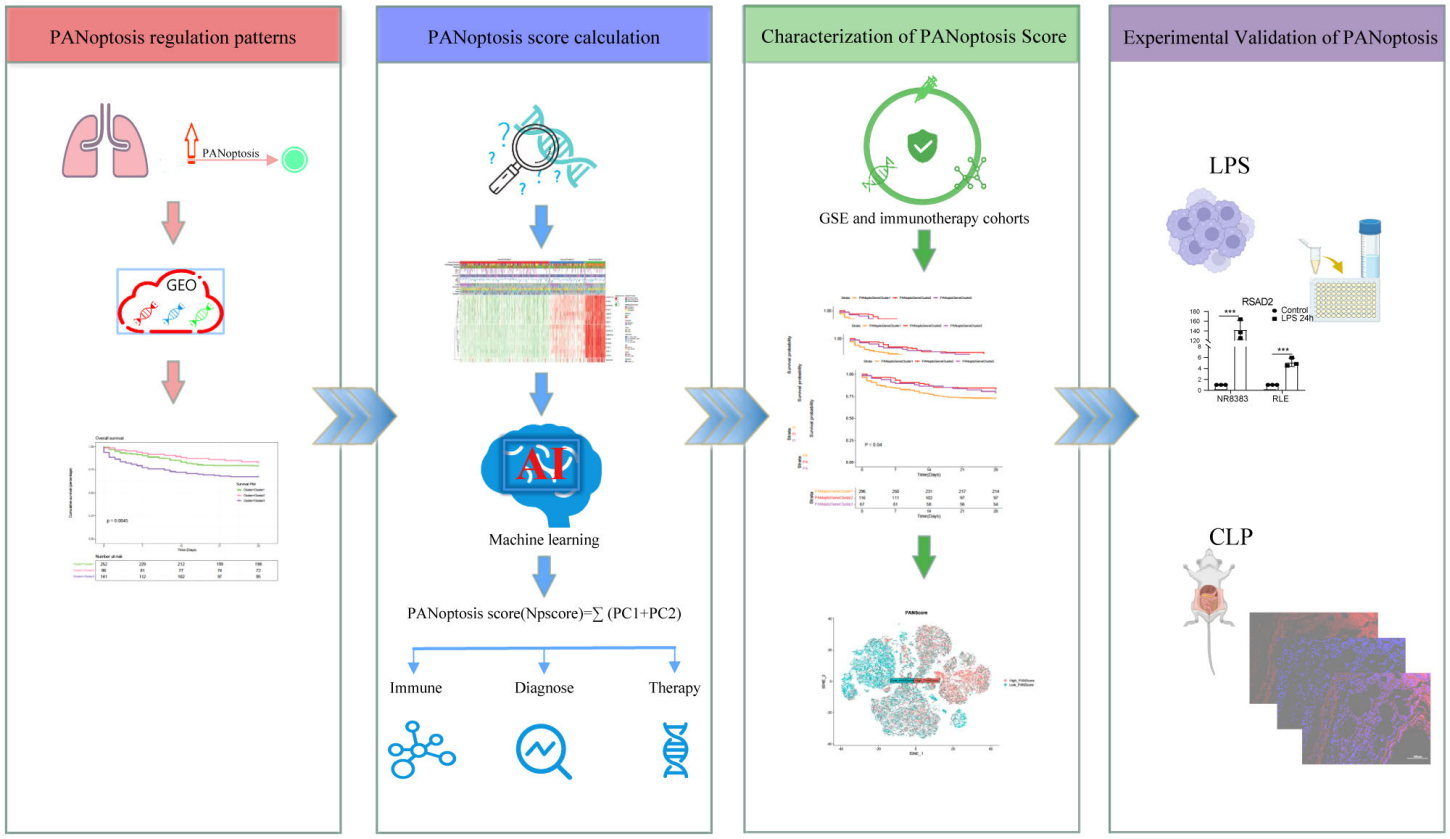
Flowchart of the study. The PANoptosis regulation pattern was obtained from the GEO dataset and the PANoptosis score was calculated with Machine learning method. And this score was further validated in bulk- and scRNA sequencing data.

The design of the study was summarized in **Figure 1**.

### 3.1 Characteristics of PANoptosis subgroups in sepsis

**Figure 2:**
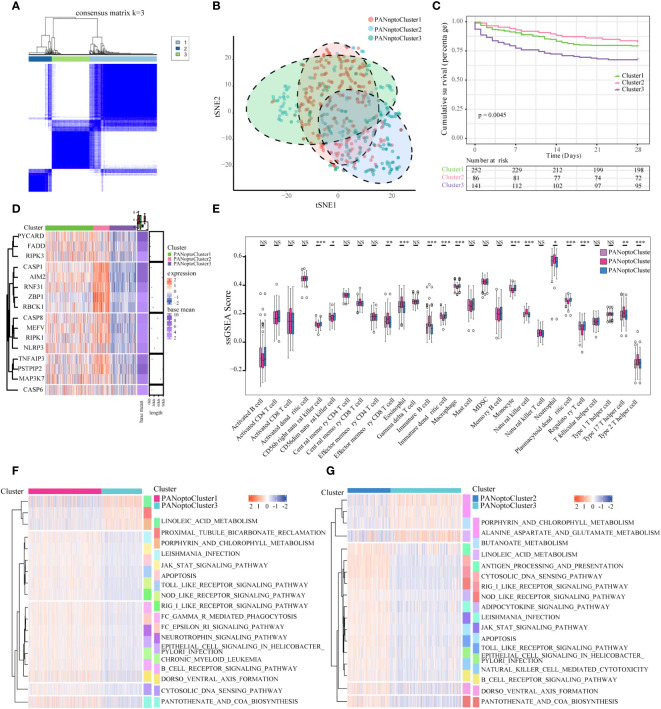
Characterization of PANoptosis subgroups in sepsis. **(A)** Consensus matrix of 16 PANoptosis factors at k = 3. **(B)** tSNE plot of PANoptosis subgroups. **(C)** Comparison of survival analysis among the three subgroups, with a statistically significant difference as a whole (P=0.0045). **(D)** Expression of PANoptosis-related genes in different subgroups, with red indicating high expression and green indicating low expression. **(E)** Immune cell infiltration characteristics of different subgroups. **(F, G)** Results of GSVA enrichment analysis showing biological pathways and PANoptosis subtypes with different activation states, including PANoptosis Cluster1 and PANoptosis Cluster2, as well as PANoptosis Cluster2 and PANoptosis Cluster3. Heatmap: red indicates activated pathways and green indicates inhibitory pathways. Different groups are used as sample annotations. P values were determined by Student’s t-test and Kruskal-Wallis test (NS P>0.05, *P < 0.05, **P < 0.01, ***P < 0.001).

Based on the expression of 16 key PANoptosis-related genes, three distinct sepsis subtypes were identified through unsupervised clustering analysis (**Figure 2A**; **Supplementary Figures 1A–E**). Optimal clustering with k=3 indicated reliable and stable differentiation into PANoptosis Cluster1, Cluster2, and Cluster3 (**Figure 2B**; **Supplementary Figure 1F**). Notably, Kaplan-Meier survival analysis demonstrated significant prognostic differences among these subtypes. Specifically, patients in PANoptosis Cluster2 exhibited a notably better survival outcome compared to those in Clusters 1 and 3 (P=0.0045, **Figure 2C**). This improved prognosis in Cluster2 might be attributed to distinct gene expression patterns, as illustrated in the heatmap (**Figure 2D**). Further, our analysis using ssGSEA highlighted substantial variations in immune cell infiltration across the subtypes. PANoptosis Cluster2 showed a markedly higher level of immune infiltration than Clusters 1 and 3 (**Figure 2E**; **Supplementary Table 2**), suggesting a possible link between immune response and patient outcomes. To deepen our understanding of the biological underpinnings of these subtypes, GSVA analysis was conducted. This revealed divergent activity in critical pathways like KEGG_CYTOSOLIC_DNA_SENSING_PATHWAY, KEGG_RIG_I_LIKE_RECEPTOR_SIGNALING_PATHWAY, and KEGG_JAK_STAT_SIGNALING_PATHWAY among the subtypes (**Figures 2F, G**; **Supplementary Figure 1G**; **Supplementary Table 3**), offering insights into the molecular mechanisms driving the observed clinical differences.

### 3.2 Construction and immune function analysis of PANoptosis genotypes

**Figure 3:**
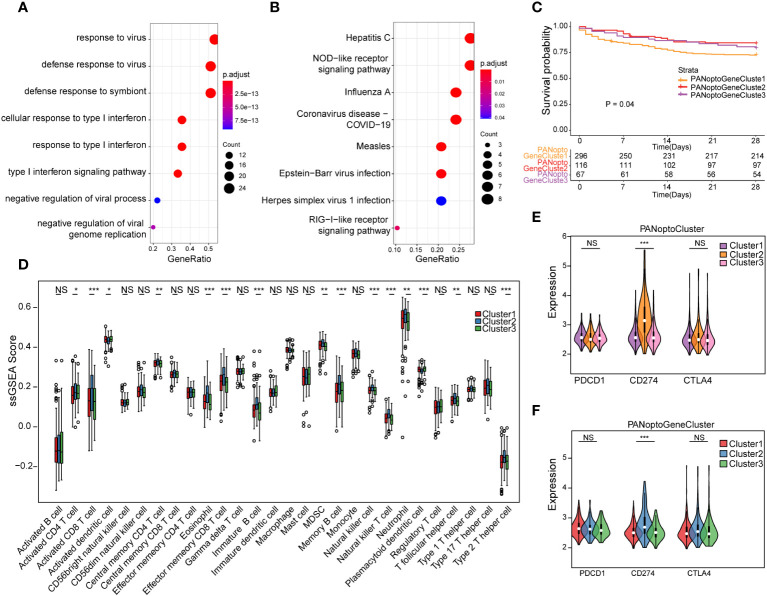
Analysis of PANoptosis genotypes. **(A)** GO analysis results of differential genes. **(B)** KEGG analysis results of differential genes. **(C)** Survival analysis comparison of the three PANoptosis gene subgroups, P=0.04, showing a statistically significant difference overall. **(D)** Immune cell infiltration characteristics of different subgroups. **(E)** Correlation analysis of PANoptoCluster subtypes with PDCD1, CD274 and CTLA4 immune checkpoints. **(F)** Correlation analysis between GeneCluster subtypes and PDCD1, CD274 and CTLA4 immune checkpoints. (NS P>0.05, *P < 0.05, **P < 0.01, ***P < 0.001).

To assess transcriptome differences between PANoptosis regulatory patterns, we performed differential analysis in these subgroups (**Supplementary Figures 2A–F**; **Supplementary Table 4**), and merging these differentially-expressed genes resulted in 48 differential genes (**Supplementary Table 5**). To analyze the functions and pathways involved in these DEGs, we used GO and KEGG analyses. In the GO analysis results, it was found that these DEGs were mainly involved in defense response to virus, defense response to symbiont, response to virus, cellular response to type I interferon, response to type I interferon, type I interferon signaling pathway, negative regulation of viral genome replication, negative regulation of viral process and other functions, mainly involved in Hepatitis C, NOD-like receptor signaling pathway, Influenza A, Measles, Coronavirus disease - COVID-19, Epstein-Barr virus infection, RIG-I-like receptor signaling pathway, Herpes simplex virus 1 infection and other signaling pathways (**Figures 3A, B**; **Supplementary Tables 6, 7**). Since PANoptosis has been reported to be related to infection immunity by many studies, the above results better reflect the functions and signaling pathways involved in PANoptosis-related differential genes.

To further extract PANoptosis-related eigengenes, we used Boruta algorithm PCA analysis to minimize the dimension of PANoptosis-related gene features to reduce noise or redundant genes, and finally obtained 16 DEGs as PANoptosis-related eigengenes (**Supplementary Table 8**). Based on these DEGs, we again used unsupervised clustering to identify different differential gene subgroups, and believed that the differences between these subgroups could better reflect the characteristics of PANoptosis subgroups. When K=3, there was the best grouping (**Supplementary Figures 3A–F**). We divided the samples into PANoptoGeneCluster1, PANoptoGeneCluster2 and PANoptoGeneCluster3 subtypes. Kaplan-Meier survival analysis showed that these subtypes were significantly different from the survival of patients, and the prognosis of the PANoptoGeneCluster2, 3 subgroup was significantly better than that of the PANoptoGeneCluster1 subgroup P=0.04 (**Figure 3C**). To clarify the differences in immune infiltration between different subgroups, we used ssGSEA to evaluate the immune cell characteristics of different subgroups (**Supplementary Table 9**), and found that the level of immune infiltration in the GeneCluster2 subgroup was overall higher than that in the GeneCluster1 and GeneCluster3 subgroups (**Figure 3D**). When we further analyzed immune checkpoints, we found that the expression of CD274 (PD-L1) was different among the three subgroups, but PDCD1 (PD-1) and CTLA4 were not different among the three subgroups (**Figures 3E, F**). These results suggest that PD-L1 may have a role in the treatment of sepsis.

### 3.3 PANoptosis score (PANscore) characteristic analysis

**Figure 4:**
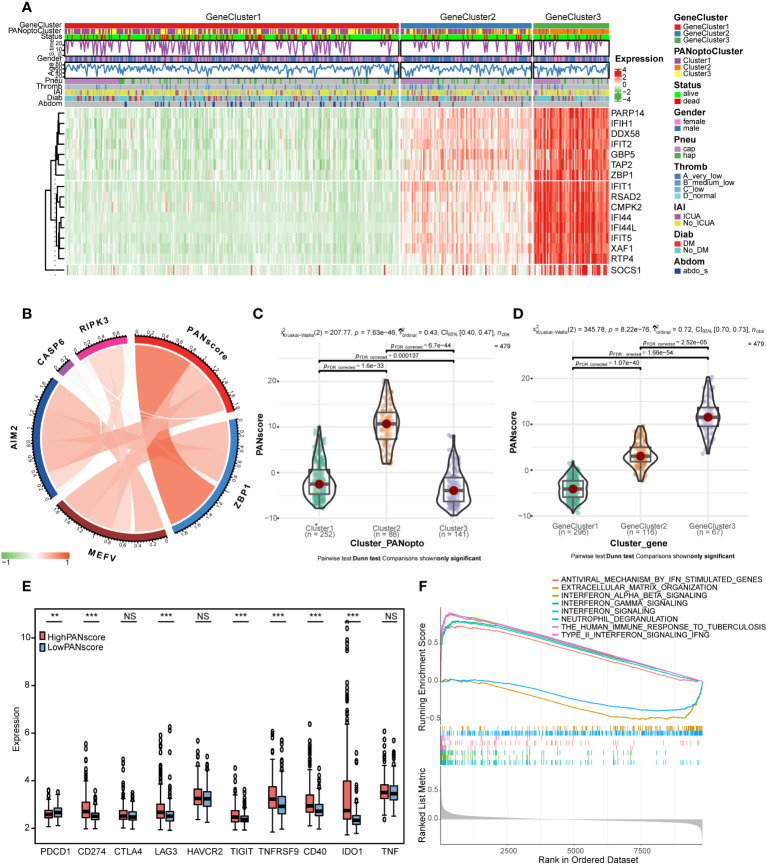
PANoptosis score (PANscore) feature analysis. **(A)** Heatmap of each subtype and clinical characteristics of patients. **(B)** Correlation analysis between PANscore and key PANoptosis genes, where red indicates positive correlation and green indicates negative correlation. **(C)** PANscore comparison of different PANoptosis subgroups. **(D)** PANscore comparison of different PANoptosis gene subsets. **(E)** Expression of immune checkpoint genes in high and low PANscore groups. **(F)** GSEA analysis. (NS P>0.05, **P < 0.01, ***P < 0.001).

We constructed a heatmap based on the expression and clinical information of PANoptosis signature genes (**Figure 4A**). The survival time-line in the low-expressing GeneCluster1 subgroup is relatively dense, and contains more PANoptoCluster1 and PANoptoCluster3 subgroups. This result is consistent with the previous survival analysis. We developed the PANscore (PANoptosis score) to apply the PANoptosis regulatory pattern to each sepsis patient based on these PANoptosis-related gene signatures (**Supplementary Table 10**). The correlation ring plot showed that PANscore was significantly positively correlated with canonical PANoptosis-related genes (**Figure 4B**). Then, we analyzed the differences of PANscore in different subgroups, and PANscore had obvious statistical differences in different subgroups (**Figures 4C, D**), which suggested that PANscore can better distinguish different PANoptosis features and can be used as a potential marker.

Only the expression of PD-L1 was significantly different as immune checkpoint in PANoptosis subgroup. We re-examined the response to ICI treatment in sepsis using PANscore and found that PD-1 was more expressed in the LowPANscore subgroup, while PD-L1, LAG3, TIGIT, TNFRSF9, CD40, IDO1 were expressed in the HighPANscore subgroup (P<0.05, **Figure 4E**).

In addition, in order to further study the biological differences between PANscore subtypes, we divided the patients into a high-score group and a low-score group based on the mean value, and performed a differential analysis, setting |logFC|>0.3 and adj.P<0.05, a total of 56 DEGs were obtained (**Supplementary Figures 3G, H**; **Supplementary Table 11**). To further assess the effect of PANscore on signaling pathways, we performed GSEA using sepsis data and obtained 101 relevant pathways (FDR<0.05, |NES|>1; **Supplementary Table 12**), including REACTOME_INTERFERON_SIGNALING, REACTOME_INTERFERON_ALPHA_BETA_SIGNALING, REACTOME_INTERFERON_GAMMA_SIGNALING, WP_TYPE_II_INTERFERON_SIGNALING_IFNG, REACTOME_ANTIVIRAL_MECHANISM_BY_IFN_STIMULATED_GENES, WP_THE_HUMAN_IMMUNE_RESPONSE_TO_TUBERCULOSIS, REACTOME_NEUTROPHIL_DEGRANULATION, REACTOME_EXTRACELLULAR_MATRIX_ORGANIZATION (**Figure 4F**), these pathways are mostly related to interferon and immune response.

### 3.4 PANoptosis score (PANscore) and clinical characteristics of sepsis

**Figure 5:**
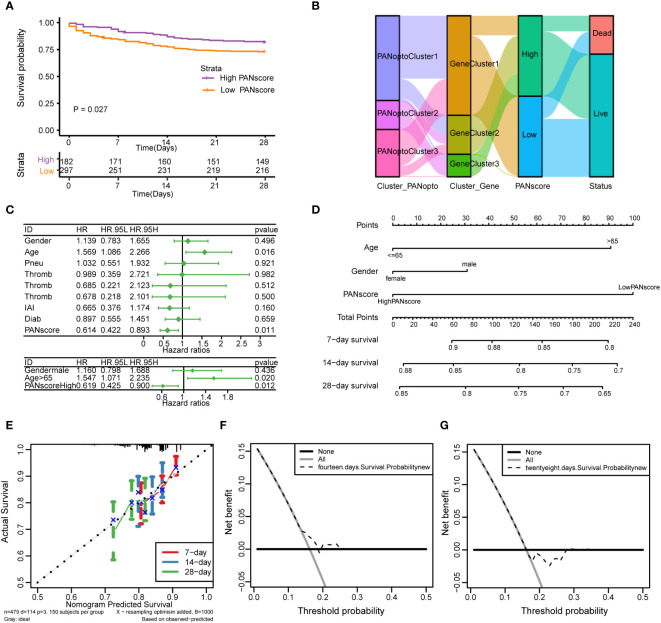
Analysis of PANoptosis score (PANscore) and clinical features of sepsis. **(A)** Survival curves show that patients with high PANscore have a better prognosis than patients with low scores in the sepsis dataset (p=0.027). **(B)** Sankey diagram showing the relationship between high and low PANscore, PANoptosis subgroups, PANoptosis gene subgroups, and survival status. **(C)** Forest plot showing the results of univariate Cox and multivariate Cox regressions on the sepsis dataset. **(D)** Nomogram constructed from the results of multivariate Cox regression on the sepsis dataset. **(E)** Calibration curves for the prognostic model at 7-day, 14-day and 28-day time point. **(F, G)** DCA curves of 14-day and 28-day prognostic models.

Based on the previously obtained PANscore for each sample, we divided patients in the sepsis dataset into high- and low-score groups according to the mean PANscore and compared their survival differences. As shown in **Figure 5A**, the prognosis of the high-score group was significantly better than that of the low-score group, P=0.027, and the difference was statistically significant. Combined with the previous analysis results, we plotted the relationship between PANoptosis score and PANoptosis regulatory subgroup, PANoptosis differential gene subgroup and survival status by Sankey plot (**Figure 5B**). Next, we identified independent prognostic factors for sepsis by univariate cox regression and multivariate cox regression, and found that age and PANscore were independent prognostic factors for sepsis (**Figure 5C**; **Supplementary Table 13**). We then drew a nomogram based on the results of multivariate cox regression (**Figure 5D**). The calibration curves of 7, 14, and 28 days (**Figure 5E**) and the evaluation of DCA curves of 14 days and 28 days (**Figures 5F, G**) all suggest that the model has a potential prognostic value.

**Figure 6:**
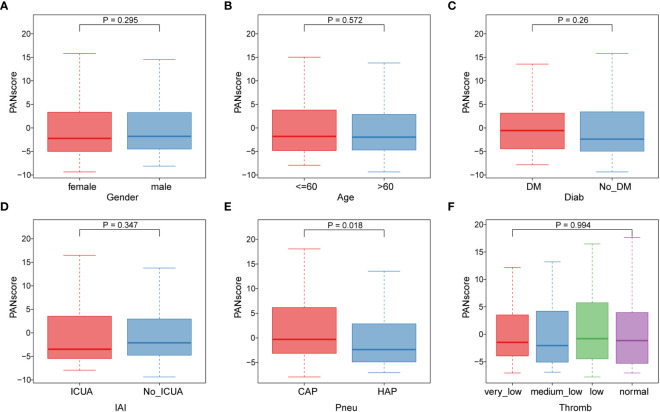
Association of PANoptosis score with different clinical features. **(A)** Comparison of gender between high and low score groups. **(B)** Comparison of age between high and low scoring groups. **(C-F)** Comparison of high and low score group stages, including diabetes **(C)**, ICU-acquired infection **(D)**, pneumonia category **(E)**, and thrombocytopenia category **(F)**.

Subsequently, we analyzed the correlation of different clinical features with PANscore. As shown in **Figure 6**, no significant differences in PANoptosis scores were found in comparisons of gender (**Figure 6A**), age (**Figure 6B**), diabetes (**Figure 6C**), ICU-acquired infection (**Figure 6D**), thrombocytopenia (**Figure 6F**). Only in the distinction of pneumonia, the PANscore of community-acquired pneumonia was found to be higher and statistically significant than that of hospital-acquired pneumonia (**Figure 6E**). This suggests that nosocomial pneumonia is associated with a low PANscore, which is also clinically consistent with sepsis.

### 3.5 Analysis of single-cell sequencing data

**Figure 7:**
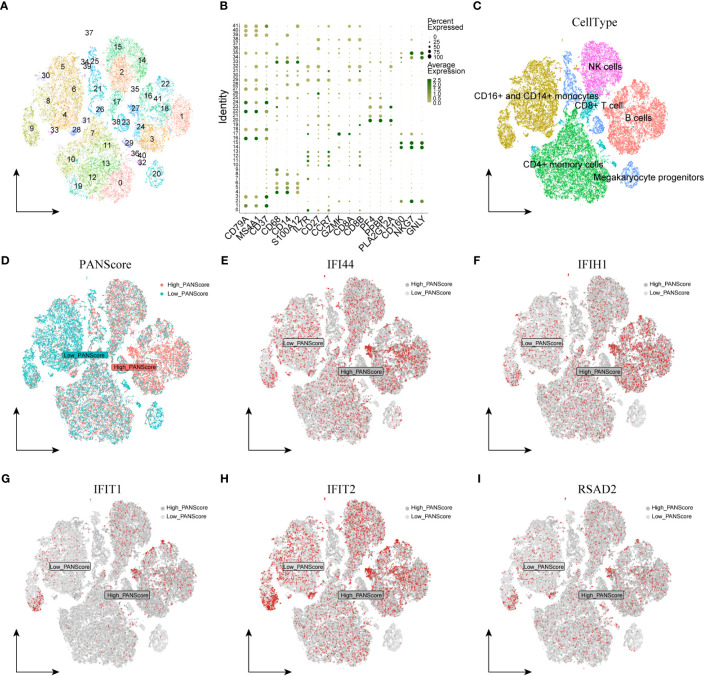
Single-cell sequencing analysis and cellular localization of PANscore signature genes. **(A)** Dimensionality reduction cluster analysis; all cells from 10 samples were clustered into 41 clusters. **(B)** Expression of cell surface marker genes. **(C)** Cells are marked as B cells, CD16+ and CD14+ monocytes, CD4+ memory cells, CD8+ T cells, Megakaryocyte progenitors, and NK cells according to the surface marder genes of different cell types. **(D)** Percentage of PANscore signature genes in each cell. Cells were divided into high PANscore cells and low PANscore cells. E-I. Localization of IFI44 **(E)**, IFIH1 **(F)**, IFIT1 **(G)**, IFIT2 **(H)** and RSAD2 **(I)** in cells.

We first performed quality control on the single-cell dataset, limiting the percentage of mitochondrial genes and erythrocyte genes to ensure the reliability of cell samples (**Supplementary Figure 4A**). All cells were then clustered into 41 clusters by the KNN clustering algorithm (**Figure 7A**). Based on the surface marker genes of different cell types (**Supplementary Table 14**), we observed their expression in different clusters (**Figure 7B**), and 6 cell types were finally identified: B cells, CD16+/CD14+ monocytes, CD4+ memory cells, CD8+ T cells, Megakaryocyte progenitors, NK cells (**Figure 7C**). To evaluate the difference of PANscore in each cell, we used the PercentageFeatureSet function to input 16 PANscore-related feature genes, and finally obtained the percentage of PANscore-related feature genes in each cell. The cells were divided into low PANscore and high PANscore according to the ratio of the median PANscore-related characteristic genes. The tSNE plot showed that high PANscore relatively accumulated in B cells, while low PANscore relatively accumulated in CD16+ and CD14+ monocytes and Megakaryocyte progenitors (**Figure 7D**).

### 3.6 Cellular localization of PANscore signature genes

We explored the expression of model genes in different cell types by single-cell sequencing analysis. The expression of PANoptosis-related genes IFI44, IFIH1, RSAD2, IFIT1, and IFIT2 varied across the immune cells. IFI44 and IFIH1 were specifically expressed in cell populations with a high PANScore, potentially linking these genes to active immune responses in B cells and implicating them in the pathophysiology of sepsis. IFIT1 and IFIT2, known for their antiviral properties, exhibited broad expression across various cell types, indicating their involvement may transcend beyond viral defense mechanisms to broader immune regulatory roles within the context of sepsis. RSAD2, a gene associated with antiviral responses, displayed a more restrained expression profile, suggesting its involvement in PANoptosis might be context-dependent within the immune cells of sepsis (**Figures 7E–I**). ZBP1, XAF1, IFI44L, SOCS1, and PARP14 were relatively high in HighPANscore cells. RTP4, DDX58, IFIT5, GBP5, CMPK2, and TAP2 were more evenly distributed in individual cells, but CMPK2, GBP5, and IFIT5 were also relatively high in cells of HighPANscore (**Supplementary Figures 4B–L**).

### 3.7 Expression of PANscore signature genes in sepsis and corresponding immunofluorescence validation in animal models

**Figure 8:**
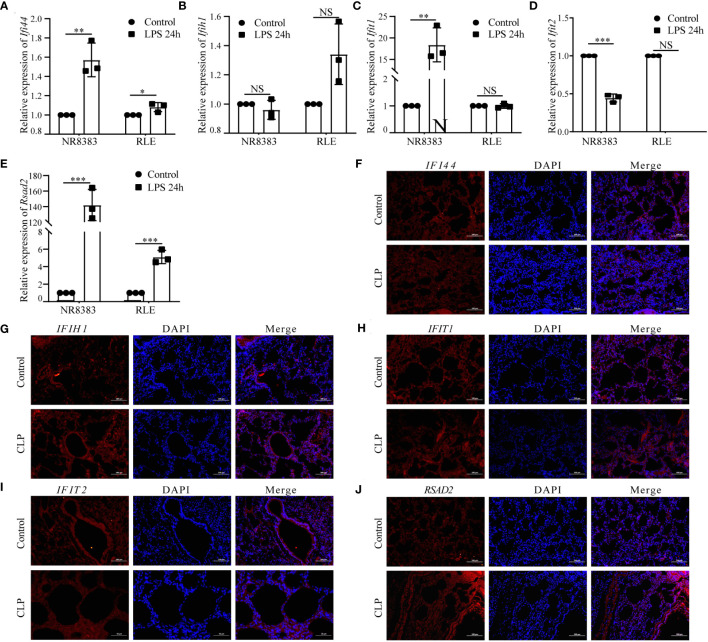
Analysis of Hub PANoptosis Genes: mRNA Expression in NR8383 and RLE Cells and Immunofluorescence in Animal Tissue. **(A–E)** mRNA expression levels of *Ifi44*, *Ifih1*, *Ifit1*, *Ifit2*, and *Rsad2* in NR8383 and RLE cells post-LPS treatment. **(F–J)** Immunofluorescence staining for *IFI44*, *IFIH1*, *IFIT1*, *IFIT2*, and *RSAD2* in lung tissue sections from the animal model. (NS P>0.05, *P < 0.05, **P < 0.01, ***P < 0.001).

We initially validated the mRNA expression of these hub genes in two cell lines. In the NR8383 cells, gene expression of *Zbp1*, *Xaf1*, *Ifi44l*, *Socs1*, *Cmpk2*, *Gbp5*, *Rtp4*, *Ifi44*, *Ifit1*, and *Rsad2* were observed to be elevated after 24h of treatment with LPS. Conversely, expression of *Tap2*, *Parp14*, and *Ifit2* diminished in these cells. In the RLE cells, gene expression of *Zbp1*, *Ifi44l* decreased, while that of *Tap2*, *Cmpk2*, *Ddx58*, *Rtp4*, *Ifi44*, and *Rsad2* increased post-LPS treatment (**Figures 8A–E**; **Supplementary Figures 5A–J**).

Protein level was conducted through immunofluorescence staining in lung tissue sections from a sepsis-induced animal model. We observed an upregulation in the expression of *IFI44*, *IFIH1*, *IFIT1*, *IFIT2*, and *RSAD2* in lung tissues from animals suffering from sepsis (**Figures 8F–J**). These augmented results corroborate the heterogeneous cellular response in sepsis and provide further layers of validation for the PANscore signature genes.

**Figure 9:**
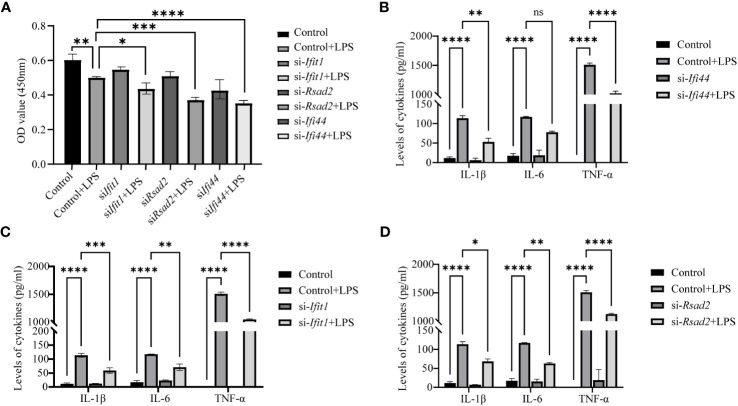
Analysis of Elisa for inflammatory markers after LPS treatment and si-RNA for hub genes. **(A)** The cell viability is assessed by OD450 nm. **(B)** The expression of IL-1β, IL-6 and TNF-α between LPS and LPS+si-*Ifi44*. **(C)** The expression of IL-1β, IL-6 and TNF-α between LPS and LPS+si-*Ifit1*. **(D)** The expression of IL-1β, IL-6 and TNF-α between LPS and LPS+si-*Rsad2*. (NS P>0.05, *P < 0.05, **P < 0.01, ***P < 0.001, ****P < 0.0001).

Further validation was performed with Elisa assay for the inflammation markers. First we found LPS can reduce the cell viability (reduced OD value), while LPS can increase the expression of IL-1β, IL-6 and TNF-α and this can be prevented by si- *Ifi44*, si- *Ifit1* and si- *Rsad2* (**Figures 9A–D**).

## 4 Discussion

Several studies have provided evidence that cell death occurring in sepsis often involves a mixed form of cell death, as cells may undergo crosstalk with each other (5, 18–20). It has been suggested that a mixed form of cell death, comprising pyroptosis, apoptosis, and necroptosis, exists in sepsis (10, 11, 21, 22). Additionally, recent studies have investigated the role of PANoptosis in various human diseases, particularly in tumors (10, 21–24). Molecular classifications of tumors and prognostic models based on genes or non-coding RNAs relevant to different forms of cell death have been reported, highlighting the potential relevance of these mechanisms in sepsis (25, 26). However, the effects of PANoptosis in sepsis remain poorly understood.

In this study, sepsis cases from the GEO dataset were divided into three distinct PANrg clusters. The cluster 2 had a better prognosis than cluster 1 and 3, which may be attributed to its higher immune infiltration, especially in effector memory CD8 T cells, immature B cells, natural killer cells, Th17 and Th2 cells. When we compared the enriched pathways between cluster 3 and cluster 2, results of GSVA and ssGSEA showed that porphyrin, alanine, glutamate, butanoate and linoleic acid metabolism reduced; while antigen processing, NK cell mediated cytotoxicity increased in cluster 2, suggesting specific metabolic and immunological profiles that may influence the prognosis in sepsis.

As infiltrating immune cells can affect the response to anti-checkpoint blockade, we therefore showed that the PD-L1 has a higher expression in both PANoptoCluster and GeneCluster 2. This indicates that higher expression of CD274 might indicate a better outcome in sepsis patients (27, 28). Furthermore, PRDEGs between two PRG clusters were also identified, and patients were classified into two distinct clusters. GO and KEGG analyses revealed that these PRDEGs were associated with response to type I interferon, NOD-like receptor signaling pathway and RIG-I-like receptor signaling pathway.

To further investigate the role of PANoptosis in sepsis, we classified clinical samples into two clusters based on the mean value of PANoptosis score. The high PANoptosis group, associated with interferon alpha and gamma signaling, neutrophil degranulation, and type II interferon signaling IFNG, highlights the significance of immune modulation in sepsis pathology (21, 29). To construct a prognostic signature, we found the high PANscore group has a better survival probability compared to the low PANscore group. Both single factor COX and multi-variable COX analysis showed that PANscore is an independent protective factor in sepsis; while age is an independent risk factor, which is further validated by the calibration curve and DCA method. This indicated that the nomogram model had high accuracy in predicting patient survival.

The correlation between the risk score and clinical characteristics was also analyzed; the PANoptosis score was only different between CAP and HAP, but not between other clinical parameters. This distinct correlation suggests a specific role of PANoptosis in different types of sepsis, potentially guiding tailored therapeutic approaches. The findings of our study can be applied to guide clinical immunotherapy in patients with sepsis and help us to further understand the effects of PANoptosis on this disease.

The expression levels of hub genes were further mapped in the single-cell RNA seq data, and showed that high PANscore relatively accumulated in B cells, while low PANscore relatively accumulated in CD16+ and CD14+ monocytes and Megakaryocyte progenitors. This highlights the differential roles of these immune cells in sepsis, influenced by their PANoptosis profiles. In our study, we observed distinct expression patterns of PANoptosis-related genes across various immune cells. The specific expression of IFI44 and IFIH1 in B cells with high PANscores underscores their pivotal role in modulating immune responses in sepsis. IFIT1 and IFIT2, although known for antiviral properties, were broadly expressed, suggesting their roles extend beyond viral defense to broader immune regulatory functions in sepsis (30, 31). Similarly, RSAD2’s constrained expression pattern in certain immune cells points to its specific involvement in PANoptosis within the sepsis context. Additionally, ZBP1, XAF1, IFI44L, SOCS1, and PARP14 were notably high in HighPANscore cells, aligning with our observations of upregulated expression of IFI44, IFIH1, IFIT1, IFIT2, and RSAD2 in lung tissues from sepsis-induced animal models. This amplifies the understanding of these genes in the immunopathology of sepsis.

The interaction of multiple cell death programs has been shown to have significant implications for the progression of sepsis. Studies have identified that PANoptosis can be induced by bacteria through Z-DNA-binding protein 1, interferon regulatory factor 1, or other risk genes (32–34). Inhibiting necroptosis has been found to normalize the apoptosis of nerve cells and protect against neuronal injury (35). Therefore, understanding the interplay between different cell death programs in sepsis is crucial to gain further insights into their cross-talk mechanisms and pathological processes in future studies.

However, our study has some limitations that warrant consideration. Primarily, the analyses were based on retrospective data from public datasets, which might introduce case selection biases and limit the generalizability of our findings. Additionally, the experimental design, primarily reliant on bioinformatic approaches, may not fully capture the complex *in vivo* interactions in sepsis. Limited molecular biology experiments were conducted, and further *in vitro* and *in vivo* experiments are necessary to validate our findings. Also, our study did not account for certain valuable clinical features, which could provide more comprehensive insights into the PANoptosis mechanism in sepsis. Therefore, clinical cases are needed to confirm our conclusions and ensure broader applicability.

In conclusion, we developed a PANoptosis-based molecular clustering and prognostic signature that can play a critical role in predicting survival and guiding clinical therapy. Our findings contribute to a better understanding of the role of PANoptosis in sepsis and could help in the development of more effective treatment strategies. Nevertheless, additional experiments and clinical cases are required to validate our results.

## Data availability statement

The original contributions presented in the study are included in the article/**Supplementary Material**. Further inquiries can be directed to the corresponding authors.

## Ethics statement

The animal study was approved by the Institutional Animal Care and Use Committee of Nanchang University. The study was conducted in accordance with the local legislation and institutional requirements.

## Author contributions

WD and PZ conceived the idea. WD designed the study. JW, SC, MD, FL and XT collected the data and performed the experiments. PZ drafted the manuscript. WD, FL and PZ reviewed and corrected the manuscript. XS and KQ supervision, project administration, funding acquisition and paper finalization. All authors contributed to the article and approved the submitted version.

## Supplementary material

Supplementary Figure 1 Sepsis PANoptosis classification. **(A–E)** Consensus matrix of 16 PANoptosis factors. **(F)** PCA principal component analysis of PANoptosis subtypes. Green circles represent the PANoptoCluster1 subgroup, pink triangles represent the PANoptoCluster2 subgroup, and purple diamonds represent the PANoptoCluster3 subgroup. **(G)** GSVA enrichment analysis showing different activation states of biological pathways and PANoptosis subtypes: PANoptosis Cluster1 and PANoptosis Cluster2.

Supplementary Figure 2 Differential analysis of PANoptosis subtypes in sepsis. **(A–C)** Heatmaps of differential analysis: PANoptoCluster1 vs PANoptoCluster2 **(A)**, PANoptoCluster1 vs PANoptoCluster3 **(B)**, PANoptoCluster2 vs PANoptoCluster3 **(C)**. Red indicates high expression, blue indicates low expression. D-E. Volcano plots for differential analysis: PANoptoCluster1 vs PANoptoCluster2 **(D)**, PANoptoCluster1 vs PANoptoCluster3 **(E)**, PANoptoCluster2 vs PANoptoCluster3 **(F)**. Light red is high expression and light green is low expression.

Supplementary Figure 3 Sepsis PANoptosis genotyping. **(A–F)** Consensus matrix of 16 PANoptosis subtype eigengenes at k = 2-4. **(G–H)** Difference analysis between high and low PANscore groups, **(G)** heat map, **(H)** volcano map. Red indicates high expression, green indicates low expression.

Supplementary Figure 4 Single-cell analysis and signature gene localization analysis. **(A)** To ensure the reliability of cell samples, the ratio of mitochondrial genes to erythrocyte genes is limited. B-L. Cellular localization of signature genes, including CMPK2 **(B)**, DDX58 **(C)**, GBP5 **(D)**, ZBP1 **(E)**, XAF1 **(F)**, SOCS1 **(G)**, PARP14 **(H)**, IFIT5 **(I)**, IFI44L **(J)**, RTP4 **(K)**, and TAP2 **(L)** in cells.

Supplementary Figure 5 Analysis of Hub PANoptosis Genes: mRNA Expression in NR8383 and RLE Cells. **(A–J)** mRNA expression levels of *Zbp1*, *Xaf1*, *Ifi44l*, *Socs1*, *Parp14*, *Cmpk2*, *Ddx58*, *Gbp5*, *Rtp4*, and *Tap2* in NR8383 and RLE cells post-LPS treatment.
