# Corrigendum: Integrated analysis of single-cell RNA-seq and chipset data unravels PANoptosis-related genes in sepsis

**DOI:** 10.3389/fimmu.2024.1415915

**Published:** 2024-04-23

**Authors:** Wei Dai, Ping Zheng, Jian Wu, Siqi Chen, Mingtao Deng, Xiangqian Tong, Fen Liu, Xiuling Shang, Kejian Qian

**Affiliations:** ^1^ Department of Intensive Care Unit, The First Affiliated Hospital of Jiangxi Medical College, Shangrao, Jiangxi, China; ^2^ Department of Clinical Medicine, Jiangxi Medical College, Shangrao, Jiangxi, China; ^3^ Department of Key Laboratory, Shanghai Pudong New Area People’s Hospital, Shanghai, China; ^4^ Department of Medical Technology, Jiangxi Medical College, Shangrao, Jiangxi, China; ^5^ Department of Intensive Care Unit, The First Affiliated Hospital of Nanchang University, Nanchang, Jiangxi, China; ^6^ The Third Department of Critical Care Medicine, Shengli Clinical Medical College of Fujian Medical University, Fujian Provincial Center for Critical Care Medicine, Fujian Provincial Key Laboratory of Critical Care Medicine, Fuzhou, China

**Keywords:** sepsis, Boruta algorithm, PANoptosis, single-cell RNA-seq, ssGSEA

In the published article, a reference was missing for the section: “We downloaded the sepsis single-cell sequencing sample GSE167363 from the GEO database and processed the data using the Seurat package, retaining cells with less than 10% mitochondrial genes, cells with a total number of genes greater than 200, and expression ranging from 200 to 12,000 and Genes expressed in at least 3 cells.”

The reference “Qiu X, Li J, Bonenfant J, Jaroszewski L et al. Dynamic changes in human single-cell transcriptional signatures during fatal sepsis. J Leukoc Biol 2021 Dec;110(6):1253-1268. PMID: 34558746” should be added.

The authors apologize for this error and state that this does not change the scientific conclusions of the article in any way. The original article has been updated.

