# Supplementary figures and images for: Integrated analysis of single-cell RNA-seq and chipset data unravels PANoptosis-related genes in sepsis
Source: Front Immunol. 2024 Jan 3;14:1247131. doi: 10.3389/fimmu.2023.1247131 (PMC10795179; doi:10.3389/fimmu.2023.1247131)

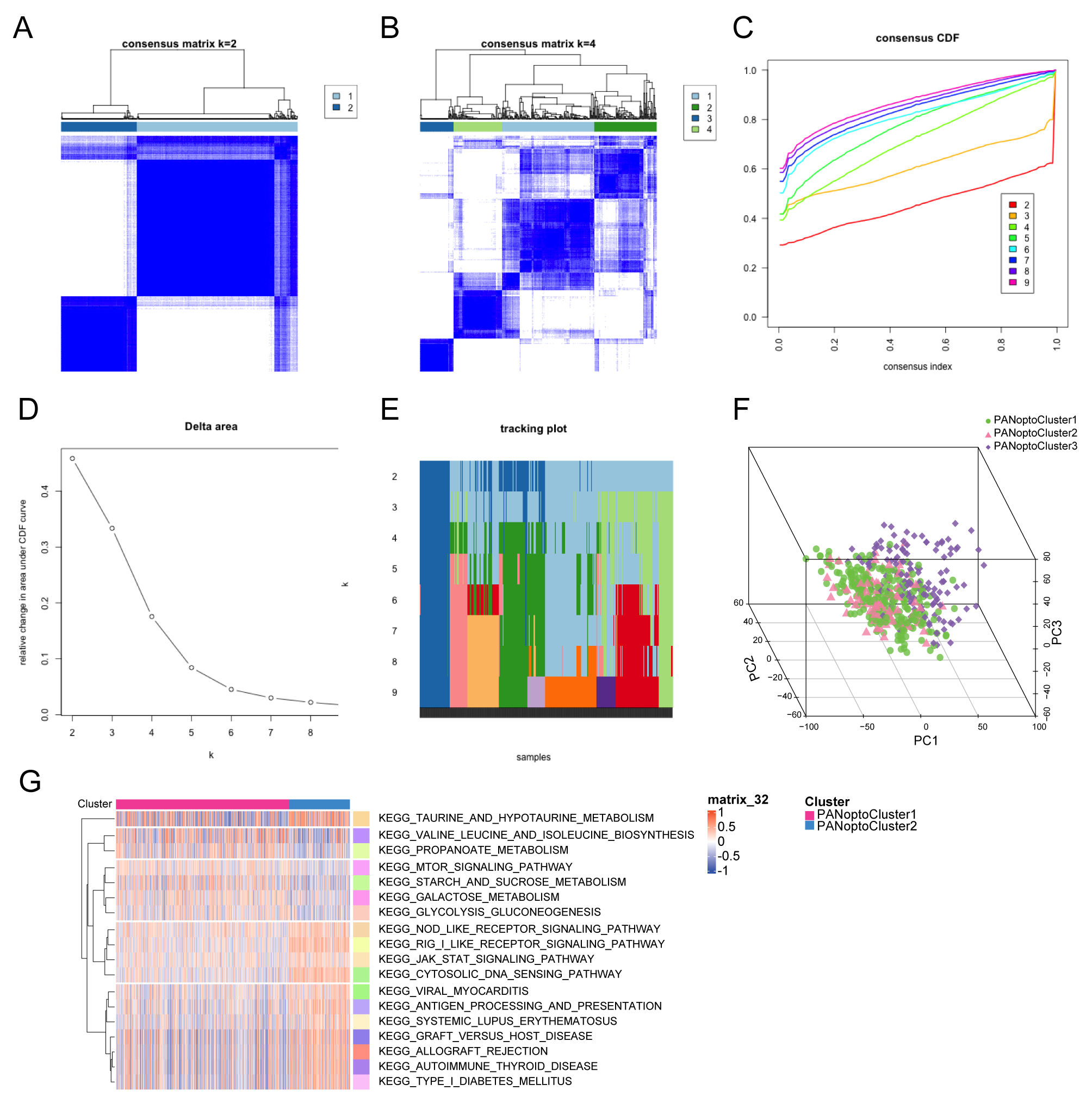

Supplement: Supplementary Figure 1 — Sepsis PANoptosis classification. (A–E) Consensus matrix of 16 PANoptosis factors. (F) PCA principal component analysis of PANoptosis subtypes. Green circles represent the PANoptoCluster1 subgroup, pink triangles represent the PANoptoCluster2 subgroup, and purple diamonds represent the PANoptoCluster3 subgroup. (G) GSVA enrichment analysis showing different activation states of biological pathways and PANoptosis subtypes: PANoptosis Cluster1 and PANoptosis Cluster2. [file Image_1.tif]

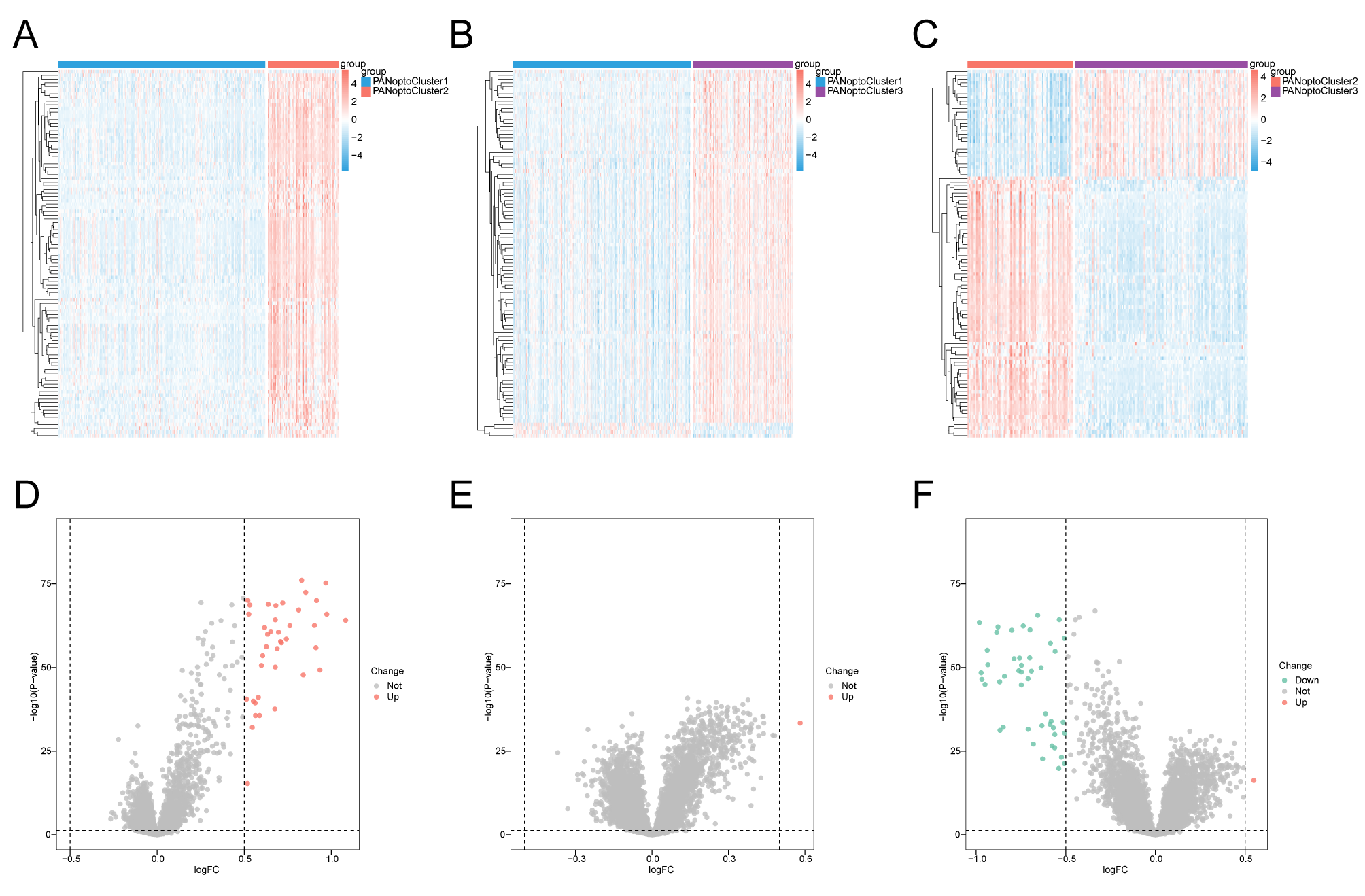

Supplement: Supplementary Figure 2 — Differential analysis of PANoptosis subtypes in sepsis. (A–C) Heatmaps of differential analysis: PANoptoCluster1 vs PANoptoCluster2 (A), PANoptoCluster1 vs PANoptoCluster3 (B), PANoptoCluster2 vs PANoptoCluster3 (C). Red indicates high expression, blue indicates low expression. D-E. Volcano plots for differential analysis: PANoptoCluster1 vs PANoptoCluster2 (D), PANoptoCluster1 vs PANoptoCluster3 (E), PANoptoCluster2 vs PANoptoCluster3 (F). Light red is high expression and light green is low expression. [file Image_2.tif]

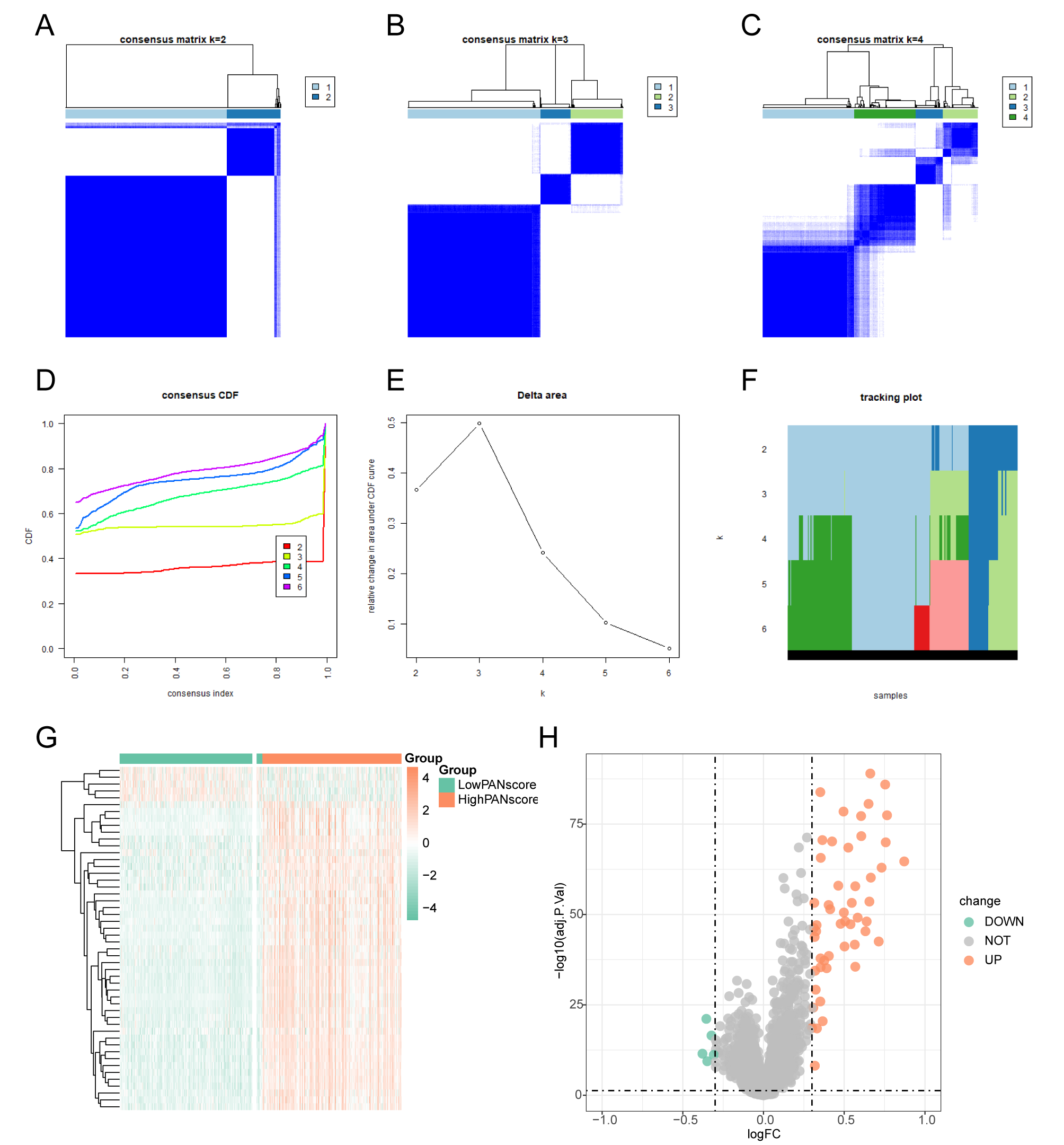

Supplement: Supplementary Figure 3 — Sepsis PANoptosis genotyping. (A–F) Consensus matrix of 16 PANoptosis subtype eigengenes at k = 2-4. (G–H) Difference analysis between high and low PANscore groups, (G) heat map, (H) volcano map. Red indicates high expression, green indicates low expression. [file Image_3.tif]

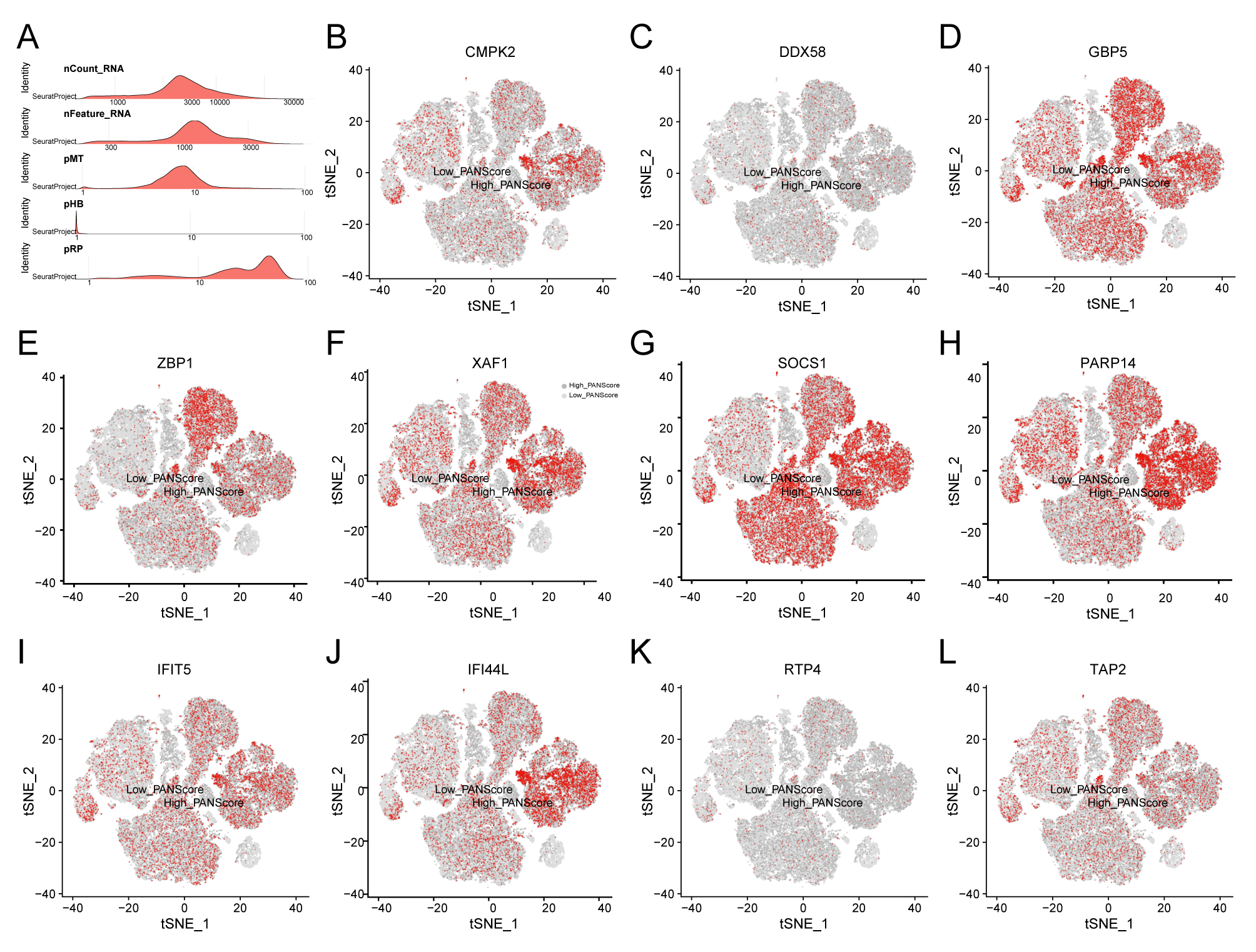

Supplement: Supplementary Figure 4 — Single-cell analysis and signature gene localization analysis. (A) To ensure the reliability of cell samples, the ratio of mitochondrial genes to erythrocyte genes is limited. B-L. Cellular localization of signature genes, including CMPK2 (B), DDX58 (C), GBP5 (D), ZBP1 (E), XAF1 (F), SOCS1 (G), PARP14 (H), IFIT5 (I), IFI44L (J), RTP4 (K), and TAP2 (L) in cells. [file Image_4.tif]

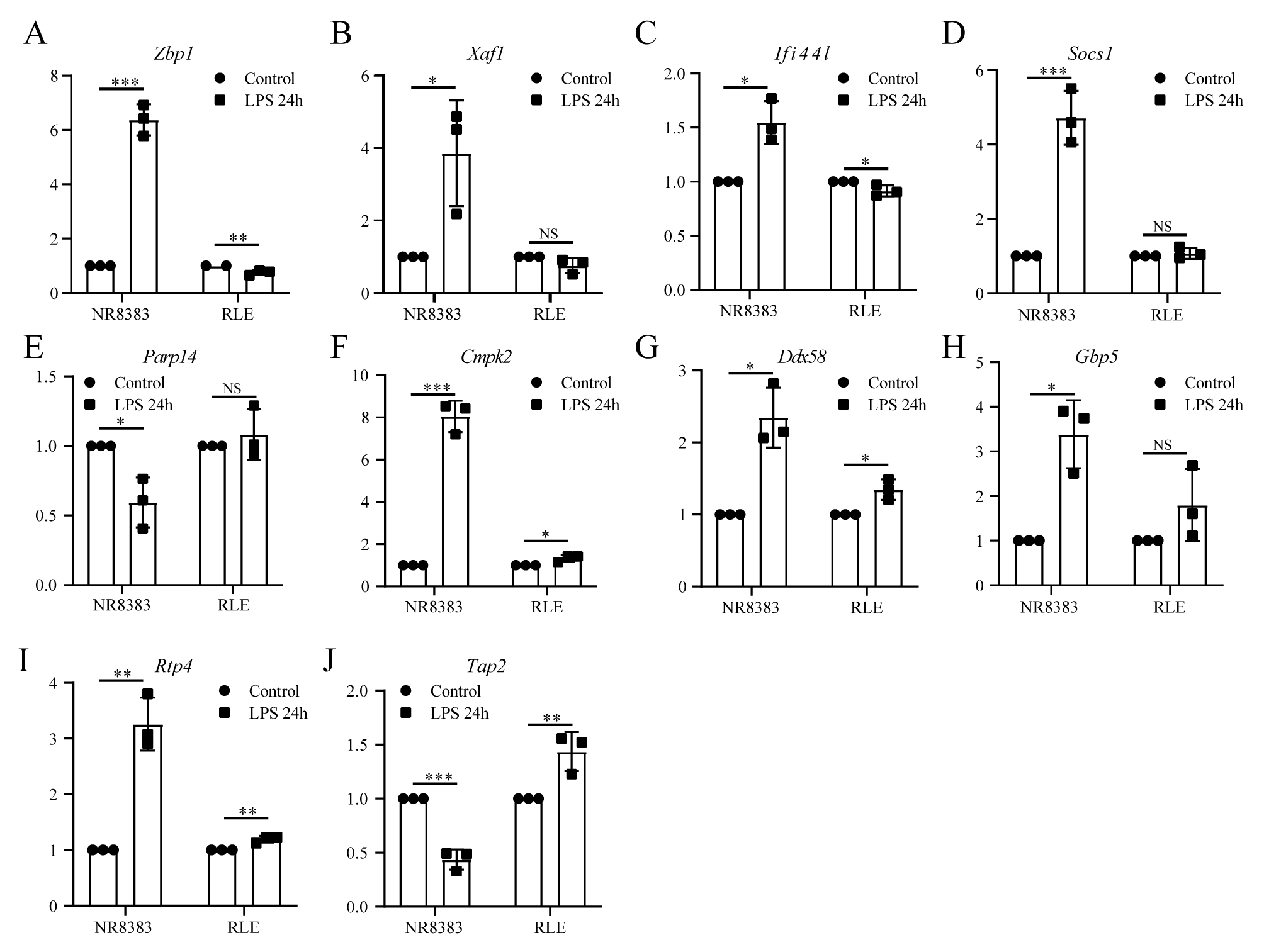

Supplement: Supplementary Figure 5 — Analysis of Hub PANoptosis Genes: mRNA Expression in NR8383 and RLE Cells. (A–J) mRNA expression levels of Zbp1, Xaf1, Ifi44l, Socs1, Parp14, Cmpk2, Ddx58, Gbp5, Rtp4, and Tap2 in NR8383 and RLE cells post-LPS treatment. [file Image_5.tif]
